# Too Useful to Avoid, Too Uneasy to Accept: Cognitive Dissonance Among Language Majors in the GenAI Era

**DOI:** 10.3390/jintelligence14100231

**Published:** 2026-09-29

**Authors:** Xiaobin Ren, Ruoxuan Wang, Ying Liang, Yuxin Song

**Affiliations:** 1 School of Foreign Languages, Guangxi University, Nanning 530004, China; 2405591025@st.gxu.edu.cn (R.W.); 2325200222@st.gxu.edu.cn (Y.L.); 2325200234@st.gxu.edu.cn (Y.S.); 2 Asia Pacific (Southeast Asia) Translation and Cross-Cultural Communication Center, Guangxi University, Nanning 530004, China

**Keywords:** generative artificial intelligence, cognitive dissonance, language majors, grounded theory, AI-mediated language education

## Abstract

This study examined the formation and regulation of cognitive dissonance among 30 undergraduates majoring in English, Chinese, Japanese, Thai, and Vietnamese through semi-structured interviews and grounded theory analysis. The findings identified Cognitive Dissonance as the core category, four dissonance-generating categories—Competence Development Disconnect, Ethical-Normative Conflict, Devaluation of Language Majors, and Educational Inertia—and one regulation-related category, Dissonance Regulation. A square-pyramid model was developed to explain the process from dissonance generation to regulation. The study shows that students’ GenAI use cannot be understood as simple acceptance or rejection, but as conflicts among beliefs, emotions, values, and behaviors around AI and language learning. It also raises concerns that early or excessive AI use may reduce opportunities for sustained practice and language competence development. This study extends the application of cognitive dissonance theory to AI-mediated language education and offers implications for language programs to strengthen AI literacy, ethical guidance, human–AI collaboration, and competence-oriented assessment.

## 1. Introduction

Generative artificial intelligence (GenAI), represented by ChatGPT-5.6 Sol, is profoundly transforming the ways university students acquire knowledge, complete academic tasks, and develop professional competence through its powerful natural language understanding and generation capabilities (Deep & Chen, 2025; Huang, 2021). This impact is particularly direct for students in language-related majors. With the support of GenAI, students can complete tasks such as text generation, translation, rewriting, polishing, and information summarization, thereby significantly improving learning efficiency (Abdelhalim et al., 2025; Woo et al., 2024). However, such use may also reduce their engagement in systematic training in listening, speaking, reading, writing, and translation, potentially creating a perceived disconnection between task completion and language competence development. Meanwhile, AI-generated content also poses challenges to academic integrity, disciplinary value, and talent cultivation models (Miao et al., 2025; Perkins & Roe, 2024). Faced with these intertwined and difficult-to-reconcile tensions, students in language-related majors may find it difficult to determine how to understand and use GenAI, and may even feel uncertain when their existing beliefs and actual behaviors become misaligned.

This uncertainty may further develop into inconsistencies among cognition, attitudes, value judgments, and actual behaviors, thereby giving rise to cognitive dissonance (Festinger, 1957). Cognitive dissonance refers to the psychological discomfort individuals experience when they simultaneously hold conflicting cognitions, attitudes, or value judgments, or when their behaviors are inconsistent with their existing beliefs. Such discomfort motivates individuals to change their behaviors, adjust their cognitions, or add new explanations in order to restore cognitive consistency. Existing research has applied cognitive dissonance theory to areas such as health behavior (Cooper & Feldman, 2020; Yakın et al., 2023), consumer decision-making (Barta et al., 2023; Wilkins et al., 2016), organizational management (Çalışkan & Gökalp, 2020; Karanika-Murray et al., 2017), and technology use (Marikyan et al., 2023; Ranjan & Mukherjee, 2026), mainly examining the formation of cognitive conflict and the ways individuals reduce dissonance. In contrast, its application in educational contexts remains relatively limited. Studies that specifically apply this theory to language education and use it to explain the cognitive conflicts and adjustment processes experienced by students in language-related majors when using GenAI are even rarer.

In addition, existing research at the intersection of GenAI and language education has mainly focused on technology acceptance and usage intention (Gong et al., 2026; Ren, 2025), writing and translation performance (Y. Wang, 2023; Woo et al., 2024), learner autonomy (Kundu & Bej, 2025; Yang, 2026), and academic integrity risks (Almanea, 2026; Uygun, 2025). Less attention has been paid to the multiple cognitive conflicts that emerge during students’ use of AI. Moreover, existing studies have often concentrated on English learners or specific language-learning tasks, particularly English writing (Ren et al., 2025; D. Wang, 2024), leaving insufficient systematic attention to the common challenges faced by students in other language-related programs, such as less commonly taught languages. Therefore, it is necessary to move beyond a narrow focus on AI use effects or attitudes toward AI and to examine, from a dynamic process perspective, how students in language-related majors perceive tensions, experience dissonance, and take action to reduce such dissonance.

Based on these considerations, this study aims to explore the formation and regulation of cognitive dissonance among students in language-related majors in the context of GenAI. Specifically, it seeks to identify the major tensions that generate cognitive dissonance, analyze its specific manifestations, and reveal how students reduce dissonance and reconstruct their developmental pathways. This study draws on semi-structured interviews with 30 university students in language-related majors and adopts grounded theory as the method of data analysis. Theoretically, it introduces cognitive dissonance theory into research on language-related education in the GenAI era, integrates issues of technological dependence, ethical norms, disciplinary value, and training into a unified process-oriented analytical framework, and extends the application of cognitive dissonance theory in AI-mediated educational contexts. Practically, this study helps illuminate the deeper psychological tensions faced by students in language-related majors when using AI, providing insights for universities to reconsider language competence development, clarify GenAI use guidelines, reform curricula and assessment systems, and strengthen professional identity and career development guidance.

## 2. Literature Review

GenAI has demonstrated a distinctly dual impact on language education. On the one hand, it can provide immediate feedback, language models, and personalized support, thereby improving task efficiency and enhancing learners’ writing, translation, and language production (Kruk & Kałużna, 2025; Song & Song, 2023). On the other hand, concerns have been raised regarding the reliability of AI-generated content, excessive reliance, reduced cognitive engagement, and academic integrity (Chan & Hu, 2023; Klimova et al., 2024; Tian et al., 2026). In response to learners’ differing perceptions of these benefits and risks, existing studies have frequently drawn on frameworks such as the Technology Acceptance Model and the Theory of Planned Behavior to examine how perceived usefulness, perceived ease of use, attitudes, and social influence shape students’ behavioral intentions and actual use of GenAI (Gong et al., 2026; Ren, 2025; Zhang, 2025). Although these studies help explain why students accept or reject GenAI, they often position learner attitudes within binary frameworks such as useful versus useless, acceptance versus rejection, or positive versus negative, making it difficult to account for the simultaneous coexistence of reliance and caution, or endorsement and skepticism.

Such ambivalence is reflected in students’ differentiated evaluations of GenAI use: they tend to accept limited assistive uses while rejecting direct content generation, yet remain uncertain about intermediate practices such as brainstorming, polishing, and partial rewriting (Johnston et al., 2024; Nelson et al., 2025). These findings suggest that GenAI use cannot be reduced to a simple distinction between compliance and misconduct; rather, it involves complex tensions among efficiency gains, grade competition, originality requirements, and judgments about acceptable use. Existing research, however, has yet to adequately explain how conflicting cognitions, value judgments, and actual behaviors coexist and interact.

Existing research on GenAI in language education remains strongly task-oriented, focusing primarily on its effects on writing (Deep & Chen, 2025; D. Wang, 2024), translation (Kruk & Kałużna, 2025; Yu, 2024), grammar correction (Chapelle, 2025; Deng & Jamaludin, 2026), and speaking practice (Kundu & Bej, 2025; X. Wang & Wen, 2025). For example, Ren et al. (2025) examined master’s students’ psychological conflicts when using ChatGPT for dissertation writing, but the study was confined to a specific academic task and mainly addressed tool adoption, perceived effectiveness, and academic risks. Although such research helps assess the value of GenAI for particular language activities, it offers limited insight into its broader implications for students’ disciplinary learning and developmental choices. For language majors, GenAI may challenge not only task performance but also the perceived value of sustained language training, professional distinctiveness, career competitiveness, and the adequacy of existing curricula for emerging competence demands.

Moreover, the literature remains heavily concentrated on English learners, particularly English writing (e.g., Sun et al., 2024; Woo et al., 2024). While this body of research is valuable, its findings may not fully capture the experiences of students in Japanese, Thai, Vietnamese, other less commonly taught language programs, or Chinese-language disciplines, which differ in training models, labor-market conditions, and perceived exposure to AI substitution. This narrow empirical focus limits understanding of whether students across language-related majors share common forms of cognitive conflict and how GenAI affects disciplinary value, educational provision, and future development more broadly.

To address the limitations of existing research, this study examines the emergence and adjustment of cognitive dissonance among students in language-related majors in the context of GenAI use. It addresses the following research questions:

RQ1: What factors contribute to cognitive dissonance among students in language-related majors when using GenAI?

RQ2: What strategies do these students adopt to reduce cognitive dissonance?

## 3. Research Design

### 3.1. Research Context

In China, students in language-related majors mainly comprise those enrolled in Chinese-language programs, particularly Chinese Language and Literature, and those in foreign-language programs, with English constituting the largest group. Other foreign-language majors include Japanese, Russian, and Spanish, as well as smaller programs in less commonly taught languages, such as Thai and Vietnamese. The rapid development of large language models has begun to reshape these students’ learning practices, everyday academic activities, and future employment prospects. While such technologies provide efficient support for writing, translation, information processing, and language practice, they also challenge traditional approaches to language competence development and raise concerns about disciplinary value, academic integrity, and career competitiveness. As a result, many students in language-related majors are experiencing growing uncertainty and confusion over how to use AI appropriately, what competencies remain essential, and how their disciplinary training can retain relevance in an AI-driven environment.

### 3.2. Research Methodology

This study adopted the Straussian tradition of grounded theory, particularly the systematic approach developed by Corbin and Strauss (2014). Grounded theory is a qualitative approach designed to generate theoretical explanations from systematically collected and analyzed data rather than to test a predetermined model or set of hypotheses. Consistent with the Straussian tradition, the present study emphasized the iterative interaction between data collection and analysis, constant comparison, theoretical sampling, and the progressive development of categories through open, axial, and selective coding. Through successive stages of coding, researchers move from participants’ accounts to initial concepts, subcategories, main categories, and ultimately an integrated theoretical framework. Although cognitive dissonance theory informed the broad focus of the study, it was not used to predetermine the coding structure or the categories developed from the data.

Grounded theory is particularly appropriate for the present study because cognitive dissonance among students in language-related majors in the context of GenAI use remains an underexplored and evolving phenomenon. Existing research has identified issues such as technological dependence, academic integrity concerns, competence erosion, and uncertainty about disciplinary value, but these issues have rarely been examined as interconnected components of a broader process. A methodology based on predefined variables may therefore overlook unexpected forms of conflict and adjustment emerging from students’ lived experiences. Grounded theory enables the study to identify, from participants’ narratives, the factors that generate cognitive dissonance, the ways students interpret these tensions, and the strategies they adopt to reduce dissonance. It also supports the construction of a process-oriented model linking the antecedents of cognitive dissonance with students’ subsequent adjustment pathways.

### 3.3. Sampling Method

This study employed theoretical sampling, a core procedure in grounded theory. Unlike probability sampling, theoretical sampling does not seek statistical representativeness; rather, participants are selected progressively according to concepts and categories emerging from ongoing data analysis. Data collection and analysis therefore proceed iteratively, with subsequent participants recruited to elaborate underdeveloped categories, explore variations, and clarify relationships among emerging concepts until theoretical saturation is reached (Corbin & Strauss, 2014).

The theoretical sampling process lasted approximately one month and resulted in in-depth interviews with 30 undergraduate students from language-related majors. During the initial stage, participants were recruited through the research team’s existing interpersonal networks. As interviews were conducted and analyzed, subsequent recruitment was guided by the emerging findings. For example, initial interviews were concentrated mainly among English majors, but preliminary analysis suggested that students’ perceptions of AI might vary across language programs and educational backgrounds. Therefore, emerging concerns prompted us to broaden subsequent recruitment to include students majoring in Thai, Vietnamese, and Japanese, as well as Chinese. With referrals from earlier participants, the research team identified students who could provide richer accounts of AI-related academic tensions, disciplinary value, and future career uncertainty, thereby enabling further development and refinement of the emerging categories. More generally, subsequent recruitment decisions were made according to the analytical needs of the emerging categories, particularly where further variation or elaboration was required, rather than according to a predetermined sampling quota.

To ensure that participants had sufficient experience of both disciplinary learning and the pressures associated with future education and employment, only third- and fourth-year undergraduates were included. The sample covered five majors: English, Chinese, Thai, Vietnamese, and Japanese. To capture variation in educational contexts and personal experiences, participants came from western, central, southern, and eastern China, as well as Southeast Asia. Recruitment and data collection for the main analytical sample continued alongside analysis until the 30 interviews generated a provisional theoretical structure comprising the major concepts, categories, and relationships. Five additional interviews were subsequently conducted to assess whether further theoretical elaboration was required and to evaluate theoretical saturation (see Section 3.6). Detailed demographic and participant characteristics of the main analytical sample are presented in Table 1.

### 3.4. Instruments and Data Collection

Semi-structured interviews were employed as the primary instrument for generating detailed textual data on students’ experiences of cognitive dissonance in the context of GenAI use. All interviews, including the interview with the participant from Southeast Asia, were conducted in Chinese. This participant was enrolled in the same courses as local Chinese students and had near-native proficiency in Chinese, enabling them to understand the interview questions and articulate nuanced personal and psychological experiences without language assistance.

An initial interview guide was developed by the research team in accordance with the research objectives and the central concerns identified in the literature. Cognitive dissonance theory and prior research served as sensitizing frameworks for the development of the interview guide rather than as predetermined analytical categories. Accordingly, the interview questions were designed to elicit participants’ experiences, perceptions, uncertainties, and responses to GenAI use in an open-ended manner. A language education specialist with a doctoral degree participated in the development of the guide and reviewed the preliminary interview questions to assess their relevance, clarity, and content validity. Following the expert review, two pilot interviews were conducted with students in language-related majors. Feedback from the pilot interviews was used to refine the wording, sequence, and scope of the questions, ensuring that they were comprehensible and capable of eliciting sufficiently detailed responses. Sample questions included: “What kinds of uncertainty have you experienced when using AI tools to support your language learning?” and “How do you perceive the impact of AI tools on language-related majors?” Follow-up questions and probes were used flexibly to clarify participants’ responses and explore emerging issues in greater depth, including concerns or experiences that had not been anticipated in advance. The flexible format of the interviews allowed participants to describe their experiences in their own terms rather than within predetermined analytical dimensions. Each interview lasted approximately 35 min.

### 3.5. Data Analysis

Data analysis followed the three-stage grounded theory coding procedure of open coding, axial coding, and selective coding (Corbin & Strauss, 2014). All 30 interviews were transcribed verbatim and imported into NVivo 11 for data management and analysis. As the interviews were conducted in Chinese, coding was performed directly on the original Chinese transcripts to preserve participants’ intended meanings and contextual nuances. All participant quotations reported in the English manuscript were translated from Chinese by a bilingual member of the research team who was involved in the data analysis. A second bilingual member of the research team checked each translated quotation against the original Chinese transcript, and any discrepancies were resolved through discussion. For culturally or linguistically ambiguous expressions, the researchers returned to the surrounding interview context and prioritized semantic and contextual equivalence rather than literal word-for-word translation. Where no direct English equivalent was available, the wording was adjusted cautiously to preserve the participant’s intended meaning.

Open and axial coding were conducted independently by two coders to enhance analytical rigor. Any discrepancies in coding were resolved through discussion, comparison of the relevant data excerpts, and refinement of the coding scheme until consensus was reached. Selective coding was then undertaken collaboratively by the two coders to integrate the main categories around the core category and clarify their relationships. Through repeated comparison and refinement across the three coding stages, a theoretical model of cognitive dissonance among students in language-related majors was ultimately developed.

Researcher reflexivity was maintained throughout the analytical process. The researchers remained attentive to the possibility that prior assumptions about GenAI and language education could shape the interpretation of the data. The pre-existing cognitive dissonance framework was therefore not used as a fixed coding scheme. Initial codes were generated from participants’ accounts, and emerging categories were retained, revised, or reconsidered according to their fit with the data rather than their consistency with prior theoretical expectations. Emerging categories were therefore repeatedly examined against participants’ original accounts, and alternative interpretations and possible overgeneralizations were considered during analytic discussions, particularly when integrating subcategories into broader categories and constructing the final model. Accounts that did not fit emerging patterns were also retained and revisited through constant comparison with supporting cases to refine the boundaries and relationships of the developing categories rather than being excluded from the analysis. This reflexive process helped keep the analysis grounded in participants’ accounts and reduced the risk of imposing preconceived interpretations on the data.

### 3.6. Theoretical Saturation

After a provisional theoretical structure had been developed from the main analytical sample of 30 participants, five additional participants were recruited specifically to assess theoretical saturation and determine whether further theoretical elaboration was required. These five interviews were not included in the main analytical sample. The additional interview transcripts were independently analyzed using the same procedures of open, axial, and selective coding, and the resulting codes and categories were compared with those derived from the main dataset. Following established grounded theory procedures (Corbin & Strauss, 2014), theoretical saturation was considered to have been reached when the additional interviews generated no new concepts or subcategories, required no substantive revision of the existing main categories, and revealed no new relationships among the categories or with the core category. The analysis met these criteria, indicating that theoretical saturation had been reached.

### 3.7. Ethics

This study was reviewed and approved by the Medical Ethics Committee of Guangxi University (Approval No. GXU-2026–070). Before each interview, participants were informed of the purpose of the study, the data collection procedures, the voluntary nature of participation, and the measures taken to protect the confidentiality of their information. Written informed consent was obtained from all participants, including permission to audio-record the interviews. Participants were also informed that they could withdraw from the study at any time without any negative consequences. All interview data were anonymized and used solely for research purposes. The recordings were subsequently transcribed verbatim and prepared for grounded theory analysis.

## 4. Results

The findings are presented in three sections corresponding to open, axial, and selective coding. Representative cases and interview excerpts are included to illustrate the analytical results and enhance the transparency of the analysis.

### 4.1. Open Coding

Open coding was used to break down the interview transcripts into meaningful analytical units and to identify concepts closely grounded in participants’ accounts (Corbin & Strauss, 2014). Through repeated reading and line-by-line comparison of the transcripts, 121 initial concepts were generated and further grouped into 22 subcategories.

For example, one participant stated, “AI is simply too useful, especially for writing and translation assignments. It saves a lot of time, and for the sake of efficiency and grades, it is really difficult not to use it.” This statement was coded as the initial concept “perceived efficiency gains from AI use” and was grouped under the subcategory “AI tool utility.” Similarly, the statement “The university still mainly looks at final grades and assignment scores. It rarely asks how the work was completed or how much AI was involved” was coded as “outcome-based assessment neglecting AI involvement” and was grouped into the subcategory “continuation of traditional teaching evaluation mechanisms.”

Owing to space constraints, Table 2 presents selected open coding results. To provide a balanced overview of the coding process, two subcategories were selected from each of the six main categories (see Section 4.2 for the axial coding results). This allows readers to trace how raw interview statements were condensed into initial concepts and then grouped into subcategories.

### 4.2. Axial Coding

Axial coding was conducted to integrate the 22 subcategories generated during open coding into broader and more abstract main categories. At this stage, the focus was not merely on grouping similar subcategories, but on identifying the underlying conceptual connections among them and clarifying how they were related to students’ cognitive dissonance in the context of GenAI use. Through repeated comparison and category refinement, six main categories were identified: Competence Development Disconnect, Ethical-Normative Conflict, Devaluation of Language Majors, Educational Inertia, Cognitive Dissonance, and Dissonance Regulation.

For example, the subcategories “Necessity of language training,” “Overdependence on AI tools,” and “Constraints on language competence development” were integrated into the main category “Competence Development Disconnect.” These subcategories together indicate that language competence still depends on sustained practice, whereas students’ frequent reliance on AI may reduce opportunities for independent language processing and, in participants’ perceptions, potentially hinder the development of language competence. Similarly, the subcategories “Normalization of AI-mediated learning,” “Continuation of traditional teaching evaluation mechanisms,” “Upgraded competence demands in the AI era,” and “Continuation of traditional training goals” were synthesized into “Educational Inertia.” This main category captures participants’ perceptions of inertia in language-related education in responding to AI-driven changes: although they perceived emerging competence demands associated with AI, they also reported that existing training goals, curricula, and assessment mechanisms remained largely grounded in traditional educational practices.

This axial coding process shows how the subcategories were elevated into more theoretically meaningful main categories, thereby providing the structural basis for the subsequent selective coding stage. The detailed axial coding results are presented in Table 3.

### 4.3. Selective Coding

Selective coding is the final stage of grounded theory analysis, in which the main categories developed through axial coding are further examined and integrated around a core category. The purpose of this stage is to construct a coherent storyline that explains the central phenomenon emerging from the data. In this study, Cognitive Dissonance was identified as the core category because it links the four dissonance-generating categories—Competence Development Disconnect, Ethical-Normative Conflict, Devaluation of Language Majors, and Educational Inertia—with students’ subsequent Dissonance Regulation.

**Figure 1:**
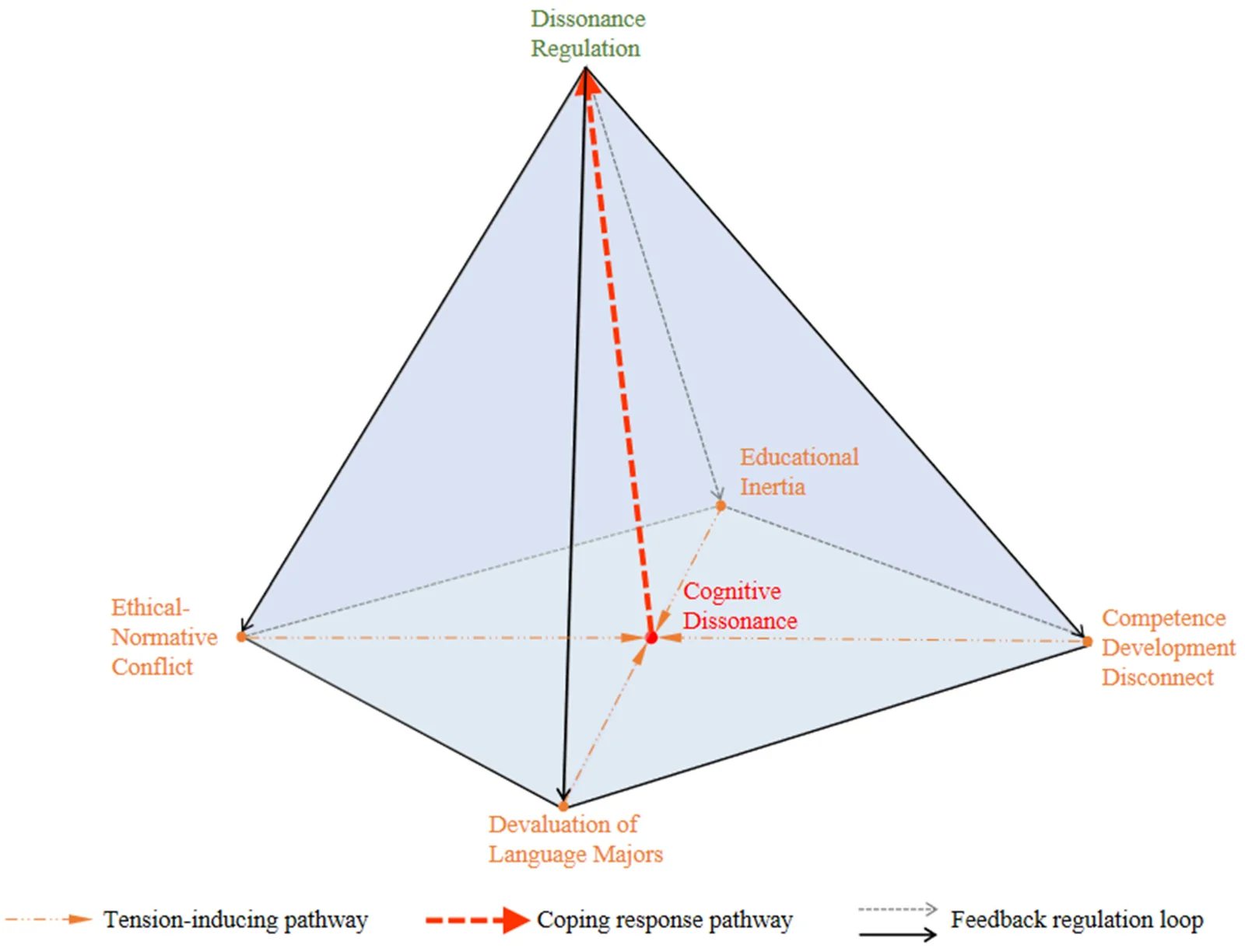
Square-pyramid model.

Based on the relationships among the categories derived through grounded theory analysis, this study developed a square-pyramid model grounded in participants’ accounts (Figure 1). The model represents three linked processes. First, the four corners of the pyramid base represent four recurrent and non-hierarchical sources of dissonance, which may operate individually or in combination across participants and converge on Cognitive Dissonance at the center of the base when experienced as incompatible cognitions, values, expectations, or cognition–behavior inconsistencies. Second, the central vertical axis from Cognitive Dissonance to the apex represents the movement from dissonance to regulation, consistent with cognitive dissonance theory’s proposition that psychological discomfort motivates efforts to restore consistency. Third, Dissonance Regulation at the apex feeds back to the sources of tension, as students attempt to reduce or reconfigure these tensions through self-defense, competence compensation, and reconstruction of developmental pathways. In this sense, the model illustrates a process from dissonance generation to dissonance regulation.

Cross-case comparison suggested that the four dissonance-generating categories did not necessarily co-occur in every participant. Rather, they appeared in different combinations across participants. For example, some accounts linked competence-related concerns with ethical-normative tension, whereas others connected concerns about disciplinary value with perceived educational inertia. The square-pyramid model therefore integrates these recurrent sources at a theoretical level and does not imply that all four categories were simultaneously present in every case.

The pathway from competence-related and ethical tensions to cognitive dissonance was clearly reflected in participants’ accounts. One participant stated, “Now whenever I have a writing or translation assignment, my first reaction is not to think by myself, but to open AI first. It gives me a version, and I revise it a little before submitting it… But after submitting it, I sometimes feel uneasy, because the result looks like mine, yet I cannot tell how much of it truly reflects my own ability.” This statement reflects participants’ perceptions of competence-related disconnection and ethical unease: students reported relying on AI to complete language tasks while questioning whether the final product represents their own competence and effort.

Another pathway concerns the combined influence of Devaluation of Language Majors and Educational Inertia. As one participant explained, “I still believe language majors should have value, but AI can already do many language-related tasks. I do not know where my irreplaceability lies… Our courses are still mainly about traditional language knowledge and final scores, while little attention is paid to how we should work with AI.” This account suggests that students’ uncertainty is associated not only with AI itself, but also with their perceived gap between changing professional demands and the responsiveness of the educational system. When students perceive that the value of language expertise is being challenged but their training has not sufficiently adapted, cognitive dissonance becomes more pronounced.

In response to such dissonance, students did not simply reject AI or remain passive. Instead, they attempted to regulate dissonance by adjusting their cognition, learning behavior, and future planning. One participant noted, “Now I do not simply ask AI for answers. I try to learn how to prompt it, how to judge where it is wrong, and how to turn its output into my own expression.” This excerpt illustrates the movement from dissonance to regulation: students seek to regain coherence by strengthening human–AI collaboration.

## 5. Discussion

The findings suggest that cognitive dissonance among students in language-related majors should not be understood simply as a personal hesitation between using and avoiding AI. This perspective also moves beyond much existing research on AI-assisted language learning that focuses mainly on technology acceptance (e.g., Gong et al., 2026; Liu et al., 2025), as it reveals students’ more complex and ambivalent attitudes toward AI. Rather than being reducible to acceptance or rejection, students’ responses may reflect one or more structural tensions in AI-mediated language education. Competence Development Disconnect, Ethical-Normative Conflict, Devaluation of Language Majors, and Educational Inertia represent recurrent sources of dissonance that may occur individually or in different combinations across students. These tensions create conditions in which students’ beliefs, values, learning practices, and future expectations become difficult to reconcile. In this sense, students’ cognitive dissonance is not merely an individual psychological response, but a structural experience shaped by the interaction of technological change, academic norms, disciplinary value, and educational systems. This finding extends the application of cognitive dissonance theory by identifying structurally situated antecedents of dissonance in the context of GenAI, suggesting that students’ dissonance may be associated not only with individual inconsistencies but also with broader tensions embedded in language education.

### 5.1. The Paradox Between AI-Assisted Efficiency and Competence Formation

Existing studies on AI-assisted language learning have often emphasized the positive role of AI in improving learning efficiency, language achievement, learner autonomy, motivation, feedback quality, and speaking performance (D. Wang, 2024; X. Wang & Wen, 2025; Wei, 2023). Participants’ accounts do not contradict these documented benefits, but they suggest that such conclusions may be incomplete when AI use is examined from the broader perspective of language competence formation rather than from isolated learning tasks. Participants expressed concern that completing a writing, translation, or presentation task with AI support may not necessarily correspond to the development of underlying linguistic competence. They also perceived that extensive reliance on AI-generated outputs could reduce opportunities for independent drafting, translating, revising, and meaning-making. Some participants further worried that early or extensive AI use in basic assignments could limit opportunities for the repetitive listening, speaking, reading, writing, and translation practice through which foundational competence is gradually developed. In this sense, AI-assisted task completion may create a perceived sense of competence that is not necessarily equivalent to underlying competence development. However, the present qualitative findings do not establish that AI use directly weakens language competence; rather, they highlight participants’ concerns about the possible effects of reduced independent practice on competence formation.

### 5.2. From Academic Misconduct to Ethical-Normative Ambiguity

Existing studies on AI ethics in language education have often focused on problematic practices such as plagiarism, fake academic writing, authenticity concerns, and academic integrity risks (Abuadas & Albikawi, 2026; Caprioglio & Paglia, 2023; Vaccino-Salvadore, 2023). While our findings also confirm these concerns, they further suggest that students’ ethical tension should not be reduced simply to intentional misconduct. Many participants were aware that AI should not replace their own work, yet they remained uncertain about where the boundary lies between acceptable assistance, excessive polishing, substantial rewriting, and direct generation. Such ambiguity becomes more salient when AI use is also associated with better assignment quality, higher grades, or competitive academic outcomes. Therefore, ethical-normative conflict arises not only from students’ violation of known academic norms, but also from the inability of existing norms to fully regulate increasingly complex AI-mediated learning practices. This finding suggests that universities should move beyond simply criticizing unethical AI use and instead accelerate the development of transparent, discipline-specific, and operational AI-use guidelines, together with assessment systems that clarify and evaluate students’ responsible use of AI.

### 5.3. Devaluation of Language Majors and Educational Inertia in the AI Era

The findings of Devaluation of Language Majors and Educational Inertia extend previous research showing that AI may reshape students’ perceptions of professional relevance, educational value, and employment risks (Borzanović et al., 2026; Liang & Zhai, 2025; Ren et al., 2026). While prior studies have mainly emphasized AI-related employment anxiety or changing labor-market demands, participants’ accounts in the present study suggest that such concerns may be intensified when students perceive language education as insufficiently responsive to AI-related changes. Students’ doubts about the value of language-related majors were therefore associated not only with their perceptions of AI-driven changes, but also with perceived inertia in existing training goals, curricula, and assessment mechanisms. This finding suggests that AI should be embedded not only in course content, but also in training objectives and assessment systems, so that language-related programs evaluate whether students can critically, ethically, and effectively use AI as part of their professional competence.

### 5.4. Dissonance Regulation

The finding of Dissonance Regulation echoes cognitive dissonance theory, which argues that individuals seek to reduce discomfort caused by inconsistent beliefs, values, or behaviors (Festinger, 1957). While previous studies have mainly highlighted AI-related anxiety, emotional fluctuation, and employment concerns among language learners (Ding & Xue, 2025; Liang & Zhai, 2025; Xin & Derakhshan, 2025), this study further identifies how students respond to such pressure. Students reduced dissonance through self-defense, competence compensation, and reconstruction of developmental pathways. This finding suggests that language education should not only recognize students’ AI-induced anxiety but also help them develop concrete strategies for AI literacy, human–AI collaboration, and future-oriented competence reconstruction.

## 6. Conclusions

This study explored cognitive dissonance among students in language-related majors in the context of GenAI use. Drawing on grounded theory analysis of interview data, the study identified Cognitive Dissonance as the core category and developed a theoretically derived square-pyramid model grounded in participants’ accounts to represent its formation and regulation. Four main categories—Competence Development Disconnect, Ethical-Normative Conflict, Devaluation of Language Majors, and Educational Inertia—emerged as recurrent sources of cognitive dissonance that could occur individually or in different combinations across participants, while Dissonance Regulation captured the ways in which students attempted to reduce such discomfort through self-defense, competence compensation, and reconstruction of developmental pathways.

The findings suggest that students’ cognitive dissonance should not be understood simply as hesitation between using and rejecting AI. Rather, it reflects deeper tensions generated by AI-mediated changes in language learning, academic norms, disciplinary value, and educational systems. In this sense, the study extends the application of cognitive dissonance theory to AI-mediated language education by highlighting structurally situated antecedents of dissonance alongside individual inconsistencies.

Practically, the study suggests that language-related programs should respond more actively to the challenges brought by GenAI. While AI tools may improve learning efficiency, language education should ensure sufficient opportunities for sustained language practice and long-term competence development, and clarify ethical boundaries for AI use. Language-related programs should also reform curricula and assessment mechanisms to include AI literacy, human–AI collaboration, critical language judgment, and interdisciplinary competence.

This study has several limitations. First, this study did not directly assess changes in students’ linguistic competence. The competence-related findings therefore represent participants’ perceptions and concerns about AI use rather than evidence of an actual decline in language competence. Second, the sample was unevenly distributed across language majors, with English majors comprising a relatively large proportion of the main analytical sample, and recruitment initially relied on the research team’s interpersonal networks and participant referrals. Although subsequent theoretical sampling was guided by the analytical needs of emerging categories rather than predetermined quotas, these recruitment procedures may have overrepresented certain experiences and limited access to perspectives outside the recruitment networks. Accordingly, the study was designed to identify cross-cutting patterns across students from several language-related majors rather than to compare individual programs, and the findings should not be interpreted as program-specific conclusions. In addition, the proposed square-pyramid model is based on qualitative interview data from a specific group of students in language-related majors and therefore should not be generalized without caution. Future research could expand the sample to different institutions, language programs, and educational levels, and could combine qualitative and quantitative methods to further examine the relationships among AI use, cognitive dissonance, competence development, and professional identity. Longitudinal research is also needed to explore how students’ dissonance and regulation strategies change as GenAI becomes more deeply embedded in language education.

## Figures and Tables

**Table 1:** Demographic characteristics of the main analytical sample (n = 30).

Demographics		F	%
Gender	Male	3	10
	Female	27	90
Grade	Third Year	22	73
	Fourth Year	8	27
Major	English	16	53
	Thai	2	7
	Vietnamese	5	17
	Japanese	4	13
	Chinese	3	10
Home place	Eastern China	2	7
	Central China	5	17
	Western China	7	23
	Southern China	15	50
	Southeast Asia	1	3

**Table 2:** Open coding results (excerpt).

Original Statements (Translated)	Initial Concepts	Subcategories
Listening requires repeated exposure, and speaking requires actually opening one’s mouth. AI can show me the answer, but it cannot make these abilities grow inside me.	Deliberate practice as the basis of language competence	Necessity of language training
Now whenever I have a writing or translation assignment, my first reaction is not to think by myself, but to open AI first. It gives me a version, and I revise it a little before submitting it.	AI replacing independent language processing	Overdependence on AI tools
Everyone is using AI, but the university and teachers rarely explain how far we can go. They only say that AI traces should not be detected, without telling us which uses are reasonable and which have crossed the line.	Lack of operational AI-use guidance	Insufficient institutional norms
AI is simply too useful, especially for writing and translation assignments. It saves a lot of time, and for the sake of efficiency and grades, it is really difficult not to use it.	Perceived efficiency gains from AI use	AI tool utility
I still believe language majors have value, because truly learning a language is not just translating sentences; it means understanding another culture, another way of expression, and another logic of thinking.	Language learning as cross-cultural understanding	Value of language majors
When I looked for internships, I found that pure translation positions had become much fewer. If I only know languages, it seems difficult to match myself with a clear job position.	Shrinking demand for translation-only positions	Declining market demand
Now whether I am making slides, revising an essay, or doing translation, I almost always use AI.	Routine reliance on AI in academic tasks	Normalization of AI-mediated learning
The university still mainly looks at final grades and assignment scores. It rarely asks how the work was completed or how much AI was involved.	Outcome-based assessment neglecting AI involvement	Continuation of traditional teaching evaluation mechanisms
I feel that language majors should still have value, but at the same time I see AI doing many language-related tasks. After studying for so many years, I am not sure where my irreplaceability lies.	Uncertainty over the irreplaceability of language expertise	Confusion about value of language majors
Sometimes AI helps me revise an assignment very well, but after submitting it, I feel uneasy. The result looks like mine, but I cannot tell how much of it truly reflects my own ability.	Unease over the authenticity of AI-assisted work	Moral unease
Now I do not simply ask AI for answers. I try to learn how to prompt it, how to judge where it is wrong, and how to turn its output into my own expression.	Developing AI literacy through critical use	Competence compensation
I do not want to rely only on one language major anymore. I am preparing to develop another direction, such as international communication, business, or data tools.	Moving from single-language competence to interdisciplinary development	Reconstruction of developmental pathways

**Table 3:** Axial coding results.

Subcategories	Main Categories	Explanation of Main Categories
Necessity of language training	Competence Development Disconnect	The perceived disconnection between AI-assisted task completion and the development of language competence.
Overdependence on AI tools		
Constraints on language competence development		
Insufficient institutional norms	Ethical-Normative Conflict	The tension between AI utility and academic norms governing originality, independence, and acceptable use.
AI tool utility		
Academic integrity requirements		
Ambiguity in the boundaries of acceptable AI use		
Value of language majors	Devaluation of Language Majors	The perceived weakening of the value, demand, and distinctiveness of language-related majors.
Declining market demand		
Weakening of professional competitiveness		
Normalization of AI-mediated learning	Educational Inertia	The perceived lag of training goals, curricula, and assessment in responding to AI-driven learning practices and competence demands.
Continuation of traditional teaching evaluation mechanisms		
Upgraded competence demands in the AI era		
Continuation of traditional training goals		
Confusion about value of language majors	Cognitive Dissonance	The psychological discomfort arising from incompatible cognitions, values, expectations, or cognition–behavior relationships concerning AI and language learning.
Moral unease		
Competence anxiety		
Uncertainty about future development		
Confusion about learning meaning		
Competence compensation	Dissonance Regulation	Strategies through which students manage and reduce cognitive dissonance by adjusting cognition, behavior, and developmental planning.
Reconstruction of developmental pathways		
Self-defense		

## Data Availability

The data presented in this study are available on request from the corresponding author. The data are not publicly available due to privacy and ethical restrictions as they consist of interview transcripts containing potentially identifiable information. Public sharing of the raw qualitative data would compromise participant confidentiality and would not be consistent with the informed consent obtained from participants. Where appropriate, only fully de-identified excerpts or aggregated coding information that cannot reasonably identify individual participants may be shared upon reasonable request.
